# Human Microbiome and Learning Healthcare Systems: Integrating Research and Precision Medicine for Inflammatory Bowel Disease

**DOI:** 10.1089/omi.2016.0185

**Published:** 2018-02-01

**Authors:** Kim H. Chuong, David R. Mack, Alain Stintzi, Kieran C. O'Doherty

**Affiliations:** ^1^ Department of Psychology, University of Guelph, Guelph, Ontario, Canada.; ^2^ Children's Hospital of Eastern Ontario (CHEO) Inflammatory Bowel Disease Centre, University of Ottawa, Ottawa, Ontario, Canada.; ^3^ Department of Pediatrics, Faculty of Medicine, University of Ottawa, Ottawa, Ontario, Canada.; ^4^ Department of Biochemistry, Microbiology and Immunology, Faculty of Medicine, University of Ottawa, Ottawa, Ontario, Canada.

**Keywords:** Big Data, biomarkers, human microbiome, inflammatory bowel disease, learning health system, precision medicine, patient engagement, trust, postgenomics medicine

## Abstract

Healthcare institutions face widespread challenges of delivering high-quality and cost-effective care, while keeping up with rapid advances in biomedical knowledge and technologies. Moreover, there is increased emphasis on developing personalized or precision medicine targeted to individuals or groups of patients who share a certain biomarker signature. Learning healthcare systems (LHS) have been proposed for integration of research and clinical practice to fill major knowledge gaps, improve care, reduce healthcare costs, and provide precision care. To date, much discussion in this context has focused on the potential of human genomic data, and not yet on human microbiome data. Rapid advances in human microbiome research suggest that profiling of, and interventions on, the human microbiome can provide substantial opportunity for improved diagnosis, therapeutics, risk management, and risk stratification. In this study, we discuss a potential role for microbiome science in LHSs. We first review the key elements of LHSs, and discuss possibilities of Big Data and patient engagement. We then consider potentials and challenges of integrating human microbiome research into clinical practice as part of an LHS. With rapid growth in human microbiome research, patient-specific microbial data will begin to contribute in important ways to precision medicine. Hence, we discuss how patient-specific microbial data can help guide therapeutic decisions and identify novel effective approaches for precision care of inflammatory bowel disease. To the best of our knowledge, this expert analysis makes an original contribution with new insights poised at the emerging intersection of LHSs, microbiome science, and postgenomics medicine.

## Introduction

Healthcare institutions in North America seek to deliver high-quality and cost-effective care in an environment of escalating costs, limited budgets, and rapid advances in biomedical knowledge and technologies. A report by the U.S. Institute of Medicine (IOM) underscores the need for a learning healthcare system (LHS) “in which science, informatics, incentives, and culture are aligned for continuous improvement and innovation, with best practices seamlessly embedded in the care process, patients and families active participants in all elements, and new knowledge captured as an integral by-product of the care experience” (IOM, 2013).

An LHS coordinates and integrates research and clinical practice to fill major knowledge gaps, improve care, reduce healthcare costs, and provide more targeted, personalized, or precision care. The growing recognition of personalized medicine or precision medicine that is targeted to individuals or groups of patients is driven by rapid development in bioinformatics, genetics, DNA sequencing, and other new technologies (Jameson and Longo, 2015). It also reflects a confluence of clinical, patient advocacy, healthcare policy, and commercial interests. In this article, we discuss the potentials and challenges of integrating the field of human microbiome research into clinical care as part of an LHS. More specifically, we highlight how patient-specific microbial data can help guide therapeutic decisions and identify novel effective approaches for precision care of inflammatory bowel disease (IBD). We outline a pediatric IBD research initiative at the University of Ottawa and Children's Hospital of Eastern Ontario (CHEO) Research Institute in Ottawa, Canada, to illustrate our points. Importantly, the analysis offered in this study also informs the global scholarship of human microbiome science and its intersections with social sciences and humanities research.

Advances in DNA sequencing and bioinformatics have enabled research beyond the human genome into the vast population of microbes in and on the human body. In 2007, the U.S. National Institutes of Health (NIH) launched the Human Microbiome Project (HMP) to characterize the microorganisms that live in and on our body across multiple body sites, including the nasal passages, oral cavity, skin, gastrointestinal tract, and urogenital tract. The Canadian Institutes of Health Research (CIHR) deployed the Canadian Microbiome Initiative to align with the HMP. In early 2008, the European Commission and China undertook a similar initiative, the Metagenomics of the Human Intestine Tract, to examine the genes of the human intestinal microbiota.

On May 13, 2016, the U.S. government announced a new National Microbiome Initiative (NMI) (White House Office of Science and Technology Policy, 2016). The NMI has three goals of supporting interdisciplinary research into microbiomes in diverse ecosystems; developing platform technologies to generate insights and share knowledge; and expanding the microbiome workforce through citizen science, public engagement, and educational opportunities.

At present, human microbiome research is moving toward a better understanding of the link between health maintenance, disease development, response to therapy, and complications of disease and outcomes with the microbial communities present in different areas of our body. Recent advances in DNA sequencing technologies have yielded important insights and suggest that patient-specific microbial data may contribute to the development of personalized or precision healthcare (Zmora et al., 2016). In particular, alterations in the intestinal microbiota have been implicated in the pathogenesis of many disorders, including IBD (Kostic et al., 2014; Sartor and Mazmanian, 2012), obesity (Koleva et al., 2015), and allergic disease (Fujimura and Lynch, 2015). Manipulation of the intestinal microbiota for disease intervention and health maintenance has captured considerable scientific, public, and commercial interest, particularly with the use of diets and probiotics (Albenberg and Wu, 2014; Hou et al., 2014; Slashinski et al., 2012; Versalovic, 2013; Winglee and Fodor, 2015; Zmora et al., 2016).

Current evidence regarding the pathogenesis of IBD and its marked heterogeneity in patient course suggests a host biological component with conditioning by environmental and intestinal microbial factors. IBD is recognized by the acute and chronic mucosal inflammation of the gastrointestinal tract. The two main subtypes are Crohn's disease (CD) and ulcerative colitis (UC), which have the highest incidence and prevalence rates in North America and parts of Europe. Moreover, IBD incidence and prevalence rates have been increasing in several regions of the world (Molodecky et al., 2012). According to Crohn's and Colitis Canada (2012), there are ∼233,000 Canadians living with IBD and the prevalence rate is nearly 0.7%, equating to more than 1 in every 150 Canadians.

There is currently no medical cure for IBD. Therapies are primarily directed to heal mucosal inflammatory responses that define the condition. Following induction of remission, the maintenance of remission is the next therapeutic goal as un-treated mucosal inflammation is associated with significant complications and morbidity. Crohn's and Colitis Canada (CCC) estimated that direct medical costs in 2012 totaled over $1.2 billion in Canada as a result of medication, hospitalization, and physician visits. Indirect costs were over $1.6 billion due to lower labor participation rates, patient out-of-pocket expenses, and short-term work absences. In addition, IBD affects quality of life and results in significant nonfinancial costs to patients and their families.

The primary goal of the pediatric IBD research initiative in the CHEO IBD clinic is to identify biomarkers for disease diagnosis and prognosis. The long-term goals are to develop point-of-care diagnostic tools and personalized therapeutic approaches using diet and antibody therapies. A number of theories have been proposed regarding the mechanisms through which the intestinal microbiota dysbiosis can activate intestinal inflammation, lead to chronicity of inflammation, alter response to therapy, and play a role in outcome (Sartor and Mazmanian, 2012). In addition, studies have examined the associations between dietary patterns in the development of IBD (Albenberg and Wu, 2014; Ananthakrishnan et al., 2014; Chapman-Kiddell et al., 2010; Costea et al., 2010; Kostic et al., 2014), and disease maintenance in new-onset pediatric CD (Amre et al., 2007). It has been demonstrated that dietary therapy can result in rapid change within a week in the intestinal microbial profile of children with CD (Lewis et al., 2015) and induction of remission of CD is possible with exclusive enteral nutrition (Ruemmele et al., 2014).

Taken together, these observations noted above suggest that functional alteration of a proinflammatory microbiome may be modified by environmental changes such as dietary intake. The CHEO IBD Centre research initiative has identified candidate biomarkers that are able to differentiate between pediatric CD and UC (Starr et al., 2016). Accurate diagnosis of IBD subtypes is important to guide appropriate and effective treatment, which can differ for CD and UC. Several studies have also been published on the development of a novel metaproteomic approach for identification and quantification of the entire human intestinal microbiota (Zhang et al., 2016a, 2016b). The approach shows a promising method to study the functions of the intestinal microbiota in health and disease and may also prove to be an approach to identify IBD subtypes and specific therapies.

Below, we first review some of the key elements that are considered to be necessary in the implementation of an LHS, as well as issues associated with harnessing the power of Big Data for integration of research and clinical care. We further discuss patient and family engagement as a key driver to fostering a learning culture in which patients and their families are informed and engaged participants. Finally, we highlight social and ethical challenges associated with integrating individual microbial data into clinical care to facilitate personalized or precision medicine.

## Key Elements of an LHS

Although the IOM conceptualization of an LHS has garnered a great deal of attention, the application of a fully integrated LHS remains largely theoretical (Psek et al., 2015). An LHS requires the active participation and collaboration of multiple and diverse stakeholders in the healthcare sector. There must be appropriate governance structures, resources for data and analytics, and approaches for evaluation and dissemination of learning initiatives. The IOM conceptualization provides a broad framework that has been much discussed and adapted to a wide range of healthcare contexts. In 2013, the U.S. National Science Foundation convened a two-day invitational workshop to identify the fundamental scientific and engineering challenges to achieving a national-scale LHS (Friedman et al., 2015). The workshop participants identified four system-level requirements, including the following: (1) an LHS trusted and valued by all stakeholders; (2) an economically sustainable and governable LHS; (3) an adaptable, self-improving, stable, certifiable, and responsive LHS; and (4) an LHS capable of engendering a virtuous cycle of health improvement.

From an ethics perspective, it has been argued that integration of research and practice in an LHS departs in important respects from the traditional distinction made between research and practice. Faden et al. (2013) propose an ethics framework that consists of seven obligations, including obligations on the part of researchers, clinicians, and healthcare administrators to respect the rights and dignity of patients and avoid imposing nonclinical risks and burdens on patients. The framework extends a similar obligation to patients to contribute to improving the quality and value of clinical care and healthcare systems.

There have been few published examples to demonstrate the practical applicability of an LHS. The majority of these examples has been developed and implemented in the American context. For example, Greene et al. (2012) presented a six-phase model for implementing a system-wide LHS at Group Health, a nonprofit healthcare system in Seattle, Washington. Their model promotes a participatory approach that involves key stakeholders in its design and implementation, timely evaluation and adjustment of system processes, and result dissemination through effective communication channels. Psek et al. (2015) described an LHS framework with nine key components based on the Geisinger Health System in Central and Northeast Pennsylvania. Some of these components include the needs for innovative partnerships, patient engagement, leadership support, and data infrastructure to capture healthcare experience and allow for real-time access to research knowledge.

According to Bernstein et al. (2015), most discussion about LHS has focused on primary care. They asserted that public health should also play a role as both a data distributor and a key stakeholder in the development of a national-scale LHS to facilitate the collaboration between public health and primary care. We provide a summary of key elements that have been explored in the academic literature (Table 1). These key elements are closely related, and the operationalization of one may have implications for another. Moreover, “for healthcare systems, one-size-fits-all solutions are rare” (Greene et al., 2012). The resources and infrastructures needed to implement and support an LHS will vary widely across different contexts. Since there are limited practical examples available, further research is needed to identify opportunities and challenges associated with implementing and supporting an LHS.

**Table 1.** Key Elements of an LHS

*Key elements*	*Description*
Patient and family engagement	An LHS recognizes and engages patients and families as active partners in the processes of learning. Strategies to engage patients and families may include the establishment of a Patient and Family Advisory Council as implemented by the Geisinger Health System (Psek et al., 2015).
Multistakeholder collaboration	An LHS is participatory and involves key stakeholders early in its design to ensure that their ideas are represented and their needs are met. A diverse set of internal and external stakeholders should be engaged, and innovative partnerships may need to be developed. Stakeholders include, but are not limited to, patients, families, relevant interest groups, identifiable communities, scientists, practitioners, staff members, and leaders in clinical, administrative, research, and data analytics areas. A nationwide LHS should engage federal agencies and public health agencies among other entities (Bernstein et al., 2015; Friedman et al., 2010).
Transparency and accountability	An LHS is open, transparent, and accountable in its operation to foster trust of all stakeholders. There is a need to rethink the traditional distinction made between clinical care and research and develop an ethics framework more suited to the priorities and needs of an LHS (Psek et al., 2015).
Adaptability	An LHS enables iterative learning and rapid adaptation to meet current and evolving healthcare needs. Scientific rigor lies at the core of an LHS to ensure the validity and credibility of research findings and their application, although there may be a need to consider and develop mechanisms to balance the potential trade-off between speed (rapidity) and accuracy (Friedman et al., 2015).
Leadership support	LHS activities should be aligned with strategic and operational goals. Senior health leaders are more willing to support an LHS model and its activities when they are aligned with existing strategic and operational goals (Psek et al., 2016). Consideration should be given to the potential tension between transformation and supporting existing strategic plans.
	Leadership support can help promote an organizational culture that embraces learning and bridge relationships across disciplinary teams, which often operate in silos. Leadership can also establish performance criteria and provide incentives and working conditions for learning activities; front-line services may be best positioned to identify gaps in healthcare and drive learning at the operational level (Psek et al., 2015).
Sustainability	An LHS is based on a sound business and governance model with strategies to enhance and sustain funding of learning activities. Financial and nonfinancial incentives should be provided to promote learning activities, particularly in clinical settings. Although an LHS is set up to improve care and lower costs, its implementation will require financial investment initially (e.g., technical and operational costs) and may compete with other organizational priorities for limited resources. (Friedman et al., 2015; Psek et al., 2015, 2016)
Data and analytics	IT infrastructure is needed to capture data at the point of care and allow for real-time assessment and knowledge generation. There should be a mechanism to protect security, privacy, and confidentiality of data and information (Psek et al., 2015).
Timely evaluation and dissemination	An LHS has the capacity to engender a continuous cycle of learning and improvement. Evaluation should be pragmatic, flexible, transparent, scalable, and timely, and should not create unnecessary or additional burdens on clinical operations or patient well-being (Psek et al., 2015). The processes of operationalizing an LHS and the performance of an LHS need to be distinguished. There may be a need to explore innovative evaluation methodologies and provide training opportunities in them. There should also be effective communication channels for timely dissemination and open discussion of research and evaluation findings with internal and external stakeholders (Greene et al., 2012; Psek et al., 2015).

LHS, learning healthcare system.

A major challenge in current healthcare involves harnessing the power of large amounts of healthcare data that are routinely collected in clinical settings. It has been argued that electronic health records (EHRs) provide a rapidly increasing source of data and hold promise as a tool for knowledge generation in healthcare (Krumholz, 2014). However, the emergence of Big Data, such as EHRs, and a range of mechanisms that enable data sharing are creating new challenges around data ownership, privacy, and confidentiality, while promoting new research and commercial opportunities (Kostkova et al., 2016). There have been a number of initiatives that involve the use of EHRs for improvement of quality of care, coordination of services, and comparative effectiveness research. For example, CancerLinQ is established by the American Society of Clinical Oncology to collect and analyze EHRs from participating oncology practices and institutions to provide real-time assessments that support clinical decisions and inform clinical practice guidelines (Schilsky et al., 2014).

ImproveCareNow (ICN) is an American pediatric network involving clinicians, researchers, and patients and their parents to improve the healthcare of children with IBD. Forrest et al. (2014) used ICN data that had been collected at each outpatient visit to evaluate the effectiveness of antitumor necrosis factor-α in the management of Crohn's disease. Their study demonstrated that using data from a pediatric LHS for comparative effectiveness research is feasible and produces valuable knowledge to important clinical questions. In Europe, TRANSFoRm 2010–2015 was an initiative that involved 21 partner organizations in ten EU member states to develop an LHS in primary care (www.transformproject.eu). It sought to develop and evaluate tools and services that integrate EHRs and other data sources to facilitate decision support for diagnosis, identification of patients eligible for research, and standardization of data elements.

The use of Big Data in an LHS raises many important questions regarding their ethical management and use in healthcare. Some of these questions include the challenges of data stewardship, data governance, transparency, and accountability to patients, providing practitioners, other stakeholders, and the public (Schilsky et al., 2014). According to Krumholz (2014), the use of Big Data requires new thinking, training, and analytic methods which, depart from traditional statistics and hypothesis testing. There is a need for researchers and funders to embrace inductive reasoning on an equal basis with deductive reasoning in scientific investigation. Big Data usage also requires the development of advanced algorithms and new analytic tools, and a careful balance of privacy and security issues with the value of data sharing. For Friedman et al. (2010), adoption and meaningful use of EHRs to achieve a nationwide LHS require collaborative participation by multiple diverse entities, including federal agencies, public health agencies, academic health centers, community medical practices, health information technology organizations, and research institutions and industry.

## Patient Engagement and Trust

As stated in the IOM report (2013), a central element of an LHS is to engage meaningfully with those it serves, including patients and their families, as well as the broader public. Patient and family engagement is recognized as a key driver in many of the aforementioned LHS examples. According to Psek et al. (2015), patient engagement can both secure the fruits of participation (e.g., data and biosamples for research and sharing) and enhance patient trust.

Effective patient engagement is particularly important for successful implementation of initiatives that involve the sharing of large amounts of patient data across multiple institutions. However, the recent failure of the care.data program in United Kingdom reveals many legal and social challenges with regard to data sharing in healthcare. In 2013, the National Health Service (NHS), England, and the Health and Social Care Information Centre (HSCIC) established care.data as a centralized data sharing system. It was set to link vast amounts of patient information for the purpose of improving healthcare and services, starting with EHRs from general practitioners and linking them to an expanded dataset of Hospital Episode Statistics. NHS England designed care.data as an “opt-out” system in which people could choose to opt out of sharing their data, rather than an “opt-in” system.

The initiative spurred considerable criticisms and was eventually suspended in 2014. The lack of public consultation, clear communication, and transparency was at the forefront of the controversy (Carter et al., 2015; Presser et al., 2015; Sterckx et al., 2016). Medical and privacy groups, as well as patients, objected to a public awareness campaign that involved sending out a leaflet without an opt-out form and adequate information about the program, including who would have access to data. A BBC poll of 860 adults estimated that over two-thirds could not recall receiving the leaflet (Vallance, 2014).

Public outcry over the lack of clarity about data safety and patient privacy deepened with news that patient data had been sold to private organizations, including insurance and drug companies (Presser et al., 2015; Sterckx et al., 2016). Moreover, the legal requirement of general practitioners to transfer patient records was perceived as breaching the confidential relationship and trust between general practitioners and patients. General practitioners were seen as being pulled in two contradicting directions, with obligations to protect their patients' data under the Data Protection Act, while having to transfer data to HSCIC under the Health and Social Care Act. A Privacy Impact Assessment of care.data by NHS England revealed that data of patients who had registered to opt out would still likely be used in research to which they had not consented (Sterckx et al., 2016). According to Carter et al. (2015, p. 5), “the experience of care.data starkly exposes an enduring truism about the limits of law: legal authority does not necessarily command social legitimacy.” In short, care.data failed to secure public trust and the status of serving public interest.

The care.data experience shows the need for strong governance over data security and meaningful stakeholder engagement to understand relevant public expectations and values in the establishment of an LHS. In their focus group study on patient attitudes toward research in clinical care, Kelley et al. (2015) found that many participants viewed research as separate from usual care and focused on the implications of research participation on their relationship with and trust in their physician. While the majority of participants expressed their support for research, there were concerns about its impact on individualized care, especially on how the use of randomization might not acknowledge their unique medical histories. For many participants, long-standing relationships with and trust in their physician played an important role in their decision to be involved in research or not. As noted by the authors, attending to physician–patient relationships and addressing patient trust could help engage patients as relevant stakeholders in the integration of research and clinical care.

Recognizing the complexity of fostering a learning healthcare environment at a local hospital level, the CHEO IBD Centre research and clinical team has utilized different methods to inform and engage patients and their families in research and discovery. Selected research articles and posters that have been presented at various medical meetings are displayed at the CHEO IBD Centre to inform youth and their parents on how biosamples are being used and about recent research discoveries. Liaison between the research team and the IBD Foundation has prompted the organization of the Youth Gut Together, an annual event in which youth, their parents, and researchers come together for education and socialization.

The 2016 event included interactive stations where graduate students and postdoctoral students demonstrated research techniques (e.g., isolating and characterizing microbial DNA), as well as tours of the research laboratories. During the event, parents and youth could learn and ask questions about research and acquire strategies to better manage IBD. It also gave youth and parents an opportunity to meet with members of the research team, and vice versa. Currently, there is no direct and objective measure of the event's impact on patient and family engagement. However, the event could potentially empower youth and parents to learn more about IBD and its management, connect with peers, and promote their interest to participate in research. For clinicians, researchers, and funders, feedback from youth and parents at the event could validate what they provide to patients and families through integrating research into clinical care, and drive the passion to continue their work.

## The Human Microbiome and Healthcare

To date, there is limited understanding of the practical and clinical relevance of most findings in human microbiome research. Despite considerable excitement in both the scientific and public communities about the medical applications of human microbiome research, most discussion regarding the integration of research and clinical care has focused on the potential of human genetic and human genomic data, and not yet on human microbiome data. Angrist and Jamal (2015) have asserted that the proliferation of large-scale genetic data as genome-wide sequencing becomes more affordable challenges the view that research should be distinct from clinical care. Whole genome and exome sequencing of people with suspected genetic conditions could be conducted on a more routine basis in clinical settings, with the results being returned to be part of patients' health records.

Conversely, genetic data generated in clinical settings could be used for research to produce generalizable knowledge, contingent upon the voluntary consent of patients. According to the authors, an LHS that takes into account individual genetic variability for the advancement of personalized medicine must have the appropriate infrastructure and mechanisms to connect patients, clinicians, researchers, and clinical laboratories to one another. These resources may include patient-facing portals, clinical and research databases, biobanks, and clinical laboratories, and their information systems.

Aronson and Rehm (2015) have proposed that EHRs are well positioned to be the technological support for storing genetic information. In the United States, the clinical sequencing exploratory research (CSER) involves several projects investigating the application of genome-wide sequencing in different clinical settings, including pediatric and adult patient care, germline testing, and developmental disabilities. Some of the CSER projects have demonstrated the potential of integrating genetic information into clinical care, including the identification of genetic alterations in prostate cancer patients (Robinson et al., 2015) and children and young adults with relapsed, refractory, or rare cancer (Mody et al., 2015) that are potentially actionable.

In 2014, CSER and Electronic Medical Records and Genomics established working groups to explore the storage and display of genomic and genetic information in EHRs (Shirt et al., 2015). Overall, the results revealed that there were substantial differences in how genetic information was documented in EHRs across participating institutions, and the needs to develop effective clinical support mechanisms and a decision support knowledge base for clinically actionable genetic information.

Rapid advances in human microbiome research suggest that profiling and manipulation of the human microbiome can provide substantial opportunity for diagnosis, intervention, risk management, and risk stratification (Hawkins and O'Doherty, 2011; Zmora et al., 2016). Unlike the human genome, which remains relatively static throughout an individual's life, the human microbiome is readily modifiable by lifestyles and environmental exposures, including diet, antibiotics, and medication among others. Thus, microbial data may be more clinically actionable than human genetic data. A case example is the gut microbiota of infants, which develops differently depending on the mode of birth delivery (vaginal delivery vs. caesarean section) and feeding method (breastfeeding vs. bottle feeding) (Albenberg and Wu, 2014). The gut microbiota changes throughout infancy and early childhood according to diets, infections, and exposure to antibiotics. In a pilot study by Dominguez-Bello et al. (2016), newborns who were delivered by caesarean section and experimentally exposed to maternal vaginal fluids had enriched gut, oral, and skin microbiota that were comparable to vaginally delivered newborns.

These early findings indicate that it is possible to partially restore vaginal microbes at birth in C-section-delivered newborns, who have a microbiota that is underrepresented of vaginal bacteria and a higher risk of developing obesity, asthma, allergies, and other immune deficiencies. However, the long-term health benefits of this microbial seeding procedure remain to be determined. Research on the association between lung microbial diversity and lung functions in cystic fibrosis patients also suggests that early intervention to maintain or enhance lung microbial diversity may slow disease progression (Coburn et al., 2015; Hampton et al., 2014). As mentioned above, the CHEO IBD Centre research initiative has identified candidate biomarkers for the differentiation of IBD subtypes, which can aid in diagnosis and treatment planning (Starr et al., 2016).

While human microbiome research holds promise to potentially provide personalized or precision care, there are many challenges that still need to be addressed. Clinical interpretation of microbial data may be complicated by the fluctuation of microbial composition across individuals and over time within an individual, presence of other clinical confounders, and use of different analytical methods that may not produce the same results even for identical datasets (Zmora et al., 2016). In their assessment of federally funded microbiome research in the U.S.A, Stulberg et al. (2016) found that most participating organizations identified the needs for high-throughput and more accurate analytical methods, standardization in sample and data collection protocols, and reference databases and biorepositories. Respondents also confirmed the needs for longitudinal and functional studies, as well as interdisciplinary research and training opportunities.

Furthermore, there are many ethical and legal concerns associated with human microbiome research, which overlap with the prevailing concerns in human genetic and human genomic research. These include issues of informed consent mechanisms, return of research results and incidental findings, data sharing and patient privacy, and ownership and benefit sharing (e.g., Hawkins and O'Doherty, 2011; Hoffmann et al., 2013; McGuire et al., 2012; Rhodes et al., 2013). At present, it is uncertain whether an individual's microbial data can provide additional information that is personal and sensitive beyond genetic data. There has been some research that suggests the potential of microbial data to identify group affiliation and more personal information.

For example, Fierer et al. (2010) reported that skin-associated bacteria recovered from the surfaces of computer keyboards and mice could be used as microbial “fingerprints” to identify the individuals, which might present a valuable resource for forensic identification. Since the human microbiome is subject to modification by lifestyles and environmental conditions, questions can be raised about the practical utility of microbial fingerprints to identify an individual after a certain amount of time (Hawkins and O'Doherty, 2011). However, this does not preclude the possibility of an individual's microbial data being combined with genetic and other types of information to reveal personal and sensitive information, such as past bacterial exposures and predispositions to certain health conditions (Hawkins and O'Doherty, 2011; Hoffmann et al., 2013).

If microbial data can be used with genetic and other types of information in ways that are personally revealing, this suggests the need for careful consideration of how microbial data may be stored and integrated into clinical care as part of an LHS. Particular emphasis should be given to safeguard patient privacy and confidentiality. Moreover, patient and family engagement is imperative to enlist patients as active participants in their own care, foster trust, and promote interest in research. In the context of biobanking to advance genomic research, O'Doherty et al. (2011) have proposed an adaptive governance model in which the regulations governing the biobank are subject to ongoing input from stakeholders to allow governance to adapt and evolve to changing needs and circumstances.

The above model recognizes participants and the public as a collective body, and employs engagement strategies to gather participant input and broader public input as necessary. As indicated in Table 1, adaptability is a key element in an LHS, which must enable iterative learning and rapid adaptation to meet current and evolving healthcare needs. Furthermore, an LHS is patient oriented and recognizes patients and their families as active partners in the processes of learning among other stakeholders. An adaptive governance approach that emphasizes fostering trust and reflexivity could enable integration of microbial data into clinical care, while making room for changing circumstances, unanticipated ethical issues, and rapid scientific advances.

In an LHS, effective communication strategies are needed not only to disseminate research evidence between researchers, clinicians, and other internal stakeholders to foster a learning culture but also to inform and educate patients and families. Tools for promoting patient knowledge and helping patients become shared decision-makers in their own care need to be customized to patients' circumstances, especially their health literacy (IOM, 2013). The Youth Gut Together event presents an annual activity to engage and enhance knowledge about IBD research and care among patients and families. While the event may empower patients and families, as well as researchers, there are challenges to coordinating such an event, including a need to balance the amount of work that is required from the research and clinical team. In articulating their vision of an LHS, the IOM (2013) has also acknowledged that coordination and integration of research into real-world clinical settings may face many challenges due to the daily demands that clinical teams already have from their work environments.

## Conclusions and Expert Outlook

LHSs have been conceptualized as being able to coordinate and integrate research and clinical practice seamlessly for continuous improvement and innovation. However, there remain many challenges to the practical implementation of such a system, particularly in regard to the ethical management and use of patient data.

There is a need for strong and adaptive governance to oversee data security, transparency, accountability, sustainability, and meaningful engagement of patients, other relevant stakeholders, and the general public. An adaptive governance approach would allow for governance to change and evolve in ways that are respectful of the needs and interests of diverse stakeholders, while taking into account rapid scientific advances and unanticipated ethical issues. While there is still limited understanding of the practical and clinical relevance of most human microbiome research findings, rapid advances in the field hold promise in aiding diagnosis, risk assessment, and treatment decision-making for many disorders. We have outlined the CHEO IBD Centre research initiative to illustrate how patient-specific microbial data can contribute to therapeutic decisions and development of precision care for IBD.

As with human genetic and human genomic data, the integration of patient-specific microbial data into clinical practice warrants careful consideration of data storage, privacy, and confidentiality. Moreover, meaningful engagement of patients and their families is imperative to foster a culture of leaning and trust. To the best of our knowledge, this expert analysis makes an original contribution with new insights poised at the emerging intersection of LHSs, microbiome science, and postgenomics medicine.

## Abbreviations Used

CCC: Crohn's and Colitis Canada
CD: Crohn's disease
CHEO: Children's Hospital of Eastern Ontario
CIHR: Canadian Institutes of Health Research
CSER: clinical sequencing exploratory research
EHRs: electronic health records
HMP: Human Microbiome Project
HSCIC: Health and Social Care Information Centre
IBD: inflammatory bowel disease
ICN: ImproveCareNow
IOM: Institute of Medicine
LHS: learning healthcare system
NHS: National Health Service
NIH: National Institutes of Health
NMI: National Microbiome Initiative
UC: ulcerative colitis
